# Efficacy of metoprolol combined with torasemide in elderly patients with degenerative valvular heart disease and heart failure and its influence on NT-proBNP levels

**DOI:** 10.12669/pjms.39.4.7465

**Published:** 2023

**Authors:** Kai Ren, Jinping Feng, Xuwen Yang, Bei Ren, Yujun Guo

**Affiliations:** 1 Kai Ren Department of Cardiology, Tianjin Chest Hospital, Tianjin 300222, P.R. China; 2 Jinping Feng Department of Cardiology, Tianjin Chest Hospital, Tianjin 300222, P.R. China; 3 Xuwen Yang Department of Recovery Unit, Tianjin Chest Hospital, Tianjin 300222, P.R. China; 4 Bei Ren Department of Cardiology, Tianjin Chest Hospital, Tianjin 300222, P.R. China; 5 Yujun Guo Department of Cardiology, Tianjin Chest Hospital, Tianjin 300222, P.R. China

**Keywords:** Senile degenerative valvular heart disease, Heart failure, Metoprolol, Torasemide

## Abstract

**Objective:**

To investigate the efficacy of metoprolol combined with torasemide in elderly patients with degenerative valvular heart disease (DVHD) and heart failure and its influence on N-terminal pro-B-type natriuretic peptide (NT-proBNP) concentrations.

**Methods:**

The records of 129 DVHD patients ≥60 years old with heart failure diagnosed and treated in our hospital from January 2020 to February 2022 were retrospectively selected. According to the treatment records, 62 patients received metoprolol treatment (Control-group), and 67 patients received metoprolol combined with torasemide treatment (Observation-group). The changes in cardiac function, NT-proBNP and inflammatory factor concentrations and clinical efficacy were analyzed and compared between the two groups before and after treatment.

**Results:**

After treatment, left ventricular ejection fraction (LVEF), left ventricular end-diastolic diameter (LVEDD) and the mitral ratio of peak early to late diastolic filling velocity (E/A) were lower, with a greater decrease in the Observation-group compared to the Control-group (*P*<0.05). After treatment, NT-proBNP and interleukin-1beta (IL-1β), tumor necrosis factor-alpha (TNF-α), interleukin 6 (IL-6) concentrations were lower in both groups, with a greater decrease in the Observation-group compared to the Control-group (*P*<0.05). The total clinical efficacy of the Observation-group was significantly higher than the Control-group (*P*<0.05). There was no significant difference in the total incidence of adverse drug reactions between the two groups (*P*>0.05).

**Conclusion:**

Metoprolol combined with torasemide has a significant therapeutic effect on DVHD patients with heart failure. This drug combination improved cardiac function, reduced NT-proBNP concentrations and has good safety.

## INTRODUCTION

Degenerative valvular heart disease (DVHD), also known as senile calcific valvular disease, is manifested as valve leaflet thickening, sclerosis, calcification, and without commissure fusion.^1^ It is a unique cardiovascular disease of the elderly, and its incidence is only lower than coronary heart disease and hypertension.^2^ The prevalence of DVHD in the elderly increases with age, through changes to the heart valves and calcium deposition, and increased damage to the aortic and mitral valves. These alterations can lead to arrhythmia, heart failure, and of the heart block.^3^ At present, heart valve replacement is the main method to treat VHD. However, this surgery can result in postoperative complications and has a long recovery time, which may be difficult for elderly patients.^4^ While DVHD patients with heart failure see some improvement in symptoms through non-invasive treatments such as diuretics and digitalis, there is still valvular disease present.^3^ Therefore, it is very important to find more effective drug therapies.

Metoprolol can significantly reduce sympathetic nerve excitability, prolong ventricular diastolic period, maintain ventricular blood flow filling, increase ventricular stroke volume, slow down heart rate, effectively protect patients’ myocardial function, and improve hemodynamics and cardiac function.^5^ Torasemide is a diuretic, increases potassium and sodium excretion, improves electrolyte levels in patients with heart failure, and improves cardiac function.^6^ Compared with Furosemide, the most common used loop diuretic, torsemide has been demonstrated to have a higher bioavailability, lower mortality, less hospitalizations and readmissions.^7^,^8^ Thus we suppose that the combination of metoprolol and torasemide may have an effect for patients with DVHD and heart failure. However, studies on the combined use of metoprolol and torasemide is limited. As such, this study mainly discussed the efficacy of the combination of these two drugs in the treatment of DVHD in patients with heart failure and the impact on NT-proBNP concentrations.

## METHODS

The clinical data of 163 DVHD patients with heart failure who were treated in our hospital from January 2020 to April 2022 were retrospectively screened, and finally 129 patients were included in the study. Sixty two patients were treated with metoprolol and considered the Control-group, and 67 patients were treated with metoprolol and torasemide and considered the Observation-group.

### Inclusion criteria:

- Diagnosed with DVHD and heart failure
- NYHA is classified as Grade-II-IV
- Age ≥60 years old

### Exclusion criteria:

- Patients with congenital heart disease or serious abnormal function of liver or kidney
- History of cognitive impairment and mental illness
- Incomplete clinical data

### Ethical Approval:

This study was approved by the Medical Ethics Committee of our hospital (No: 2020YS-042-01; Date: 2020-08-26).

All patients in both groups received a low-salt and low-fat diet, controlled water and sodium intake, and received diuretic drug Furosemide (Jilin Yinhe Pharmaceutical Co., Ltd., Specification: 20mg, Approval No. H22023057). Meanwhile, angiotensin converting enzyme inhibitor (ACEI) or angiotensin II receptor blocker (ARB) was given for the treatment of heart failure. Cardiotonic drug Digoxin (Sanofi Hangzhou Pharmaceutical Co., Ltd., Specification: 0.25mg, Approval No. H33021738) was also given when necessary. Depending on the health of the patient, an oxygen mask was used where necessary.

Patients in the Control-group received metoprolol tartrate injection as the conventional treatment. Specifically, 15mL of drug was added to 500mL of 0.9% sodium chloride which was given to patients through an intravenous drip two to three times a day. Patients in the Observation-group received metoprolol combined with torasemide. Metoprolol was prepared as previously described in the Control-group, while 250 mL of torasemide was added to 250mL of 0.9% sodium chloride solution which was given to patients through an intravenous drip once a day. The initial dose of torasemide was 10mg/day and was provided to patients on an individual basis. Specifically, the dose could be increased to 20 mg/day with the maximum dose not exceeding 40mg/day based on the severity of patient’s condition. The treatment time of both groups was one week. During the treatment, patients were instructed to stay in bed, maintain a low salt and low-fat diet, and reasonably control drinking water.

Data was collected before and after treatment. For all patients, LVEDD, LVEF and E/A were measured using apical four chamber color Doppler ultrasound (GE, USA). The concentrations of NT-proBNP, IL-1β, TNF-α and IL-6 were also analyzed. Whole blood (5mL) from the antecubital vein was taken in the morning. Upon collection, 40mL of disodium ethylenediaminetetraacetate and 40μL of aprotinin were immediately injected into the test tube, mixed well, and centrifuged for 15 minutes at 4°C and 3000 r/minute. The plasma was analyzed on the same day or stored in a refrigerator at - 80°C for future testing. The level of NT-proBNP in plasma was detected by automatic immunochemiluminescence (Siemens ADVIACentaur XP). Reagents and instruments for plasma IL-1, TNF - and IL-6 were provided by Siemens Immune 1000 [W2], Beijing North Biotechnology Research Institute and Backman Coulter of the United States, respectively.

### Efficacy evaluation:^9^

After one week of treatment, if the relevant clinical signs and symptoms of the patient partially or completely disappeared, and the cardiac function improved to Grade-I compared with that before treatment, which was judged to be significantly effective. After treatment, if Grade-I of the patient’s relevant signs and symptoms were significantly improved compared with those before treatment, and the cardiac function was improved to Grade-I compared with those before treatment, which was determined as effective. After treatment, if the patient’s physical signs and symptoms were not significantly improved compared with those before treatment, and the cardiac function was not significantly improved, or the condition was further aggravated, so it was determined as non effective. (Number of significant cases + number of effective cases)/total number of cases × 100%=total effective rate. Any adverse reactions such as headache and dizziness were also observed.

### Statistical analysis:

Data analysis was completed using SPSS 22.0. Descriptive statistics (mean, standard deviation, count) are used to describe the classification and quantitative variables. The chi square test was used to compare discrete variables, and the student t-test was used to compare continuous variables. The t-test was used to compare cardiac function, NT-proBNP, and inflammatory factor levels between the two groups. The chi square test was used to compare the total efficacy and adverse reactions between the two groups. All statistical tests were bilateral, and when *P*<0.05, it was statistically significant.

## RESULTS

A total of 129 patients 70 males and 59 females, aged 67.9 ± 4.4 years old were enrolled in this study. There was no significant difference in gender, age, and disease duration between the two groups (*P*>0.05) (Table-I). Before treatment, there was no significant difference in cardiac function indexes between the two groups (*P*>0.05). After receiving the same course of treatment, LVEF, LVEDD and E/A of patients in the Observation-group were higher than those in the Control-group, with statistically significant differences (*P*<0.05; Table-II); Before treatment, the levels of NT-proBNP, IL-1β, TNF-α and IL-6 were not different between groups (*P*>0.05). Treatment resulted in lower plasma concentrations, with significantly lower levels in Observation-group compared to the Control-group (*P*<0.05; Table-III). The total effective rate of the Observation-group (97.01%) was significantly higher than that of the Control-group (83.87%; *P*<0.05), however, there was no significant difference in the incidence of adverse reactions between the two groups (*P*>0.05; Table-IV).

**Table-I:** Baseline characteristics of the patients.

Group	N	Male/Female (n)	Age (year)	Disease duration (year)
Control-group	62	33/29	67.74±4.37	6.60±2.70
Observation- group	67	38/29	68.04±4.50	6.63±2.60
t		0.159	-0.387	-0.064
p-Value		0.690	0.699	0.949

**Table-II:** Comparison of LVEF, LVEDD and E/A indexes between the two groups before and after treatment (*χ̅*±*S*).

Group	*n*	LVEF (%)		LVEDD (mm)		E/A	

		Before treatment	After treatment	Before treatment	After treatment	Before treatment	After treatment
Control-group	62	46.38±6.04	63.79±5.24^a^	51.74±5.28	53.98±7.02^a^	1.21±0.12	1.34±0.21^a^
Observation-group	67	46.06±5.49	69.13±4.99	52.06±5.17	59.85±6.31^a^	1.22±0.13	1.58±0.28^a^
*t*	-	0.322	-5.928^a^	-0.345	-4.966	-0.815	-5.882
*p-Value*	-	0.748	<0.001	0.731	<0.001	-0.417	<0.001

***Note:*** LVEF-left ventricular ejection fraction, LVEDD-left ventricular end-diastolic diameter, E/A-the mitral ratio of peak early to late diastolic filling velocity;

a means that compared with the same group before treatment, P<0.05.

**Table-III:** Comparison of NT-proBNP, IL-1β, TNF-α and IL-6 levels between the two groups before and after treatment (*χ̅*±*S*).

Group	n	NT-proBNP (pg/ml)		IL-1β (μg/L)		TNF-α (pmol/L)		IL-6 (ng/L)	

		Before treatment	After treatment	Before treatment	After treatment	Before treatment	After treatment	Before treatment	After treatment
Control-group	62	1850.06±159.12	527.69±91.49^a^	0.49±0.14	0.28±0.13^a^	16.63±2.91	13.20±2.82^a^	124.09±18.53	106.27±16.87^a^
Observation-group	67	1873.76±156.90	461.22±98.94^a^	0.47±0.16	0.20±0.15^a^	16.88±3.17	9.75±2.95^a^	122.18±21.26	90.97±17.80^a^
t	-	-0.851	3.952	1.060	3.002	-0.482	6.785	0.544	5.002
p-Value	-	0.396	<0.001	0.291	0.003	0.631	<0.001	0.587	<0.001

***Note:*** NT-proBNP (N-terminal pro-B-type natriuretic peptide); IL-1β (interleukin-1beta); TNF-α (tumor necrosis factor-alpha); IL-6 (interleukin 6);

a means that compared with the same group before treatment, P<0.05.

**Table-IV:** Comparison of the total effective rate and adverse reactions between the two groups [n (%)].

Group	n	Total effective rate				Adverse reactions				

		Significantly effective	Effective	Invalid	Total effective rate	Headache	Vertigo	Insomnia	Nausea	Total
Control-group	62	21 (33.87)	31 (50.00)	10 (16.13)	83.87%	2 (3.22)	1 (1.61)	1 (1.61)	1 (1.61)	5 (8.06)
Observation-group	67	32 (47.76)	33 (49.25)	2 (2.99)	97.01%	0 (0)	1 (1.49)	2 (2.99)	2 (2.99)	5 (7.46)
χ^2^	-				7.496					2.687
P	-				0.024					0.611

## DISCUSSION

This study retrospectively analyzed the clinical data of 129 elderly patients with DVHD and heart failure. The results showed that metoprolol combined with torasemide in the treatment of DVHD patients with heart failure is safe and resulted in improved cardiac function, significantly reduced NT-proBNP concentrations, which improved overall clinical efficacy. A prospective study by Cheng X et al^10^ shows that metoprolol can improve cardiac function, motor function and quality of life in Chinese patients with chronic heart failure (CHF). Clinical studies have shown that metoprolol, when used in the treatment of patients with heart disease, mainly promotes peripheral vascular dilation, improves water and sodium retention, alleviates the degree of myocardial injury, and effectively protects the myocardial function of patients by slowing down the heart rate and exerting the inhibitory effect of myocardial contraction.^11^,^12^ Although furosemide is a strong diuretic that has been used for decades, torsemide has been reported to be associated with greater bioavailability, less deaths, and lower risk of rehospitalizations.^6^,^7^

Torasemide is a sulfonylurea pyridine diuretic. It has good sodium, potassium and diuretic effects during application. It can directly act on the thick segment of the ascending branch of the Henry’s medullary loop, effectively inhibit the Na^+^/K^+^/2C^−^ carrier system, and promote increased excretion of Cl^−^, Na^+^ and water in urine. However, the drug will not have a significant impact on renal plasma flow, glomerular filtration rate, and acid-base balance in the body, Torasemide plays a significant role in improving the clinical efficacy and cardiac function of patients with heart failure.^13^,^14^ This study shows that the cardiac function of patients in the Observation-group after treatment was better than that in the Control-group, indicating that the combination of the two drugs can induce a greater improvement in cardiac function.

The results of this study showed that the levels of NT-proBNP, IL-1β, IL-6 and TNF-α in patients treated with metoprolol combined with torasemide were significantly lower than those treated with metoprolol alone. The combination of metoprolol and torasemide can play a complementary and synergistic role, effectively improve the regulation of serum cytokine level, reduce the inflammatory reaction of patients, and help improve the overall efficacy of patients. At present, clinical studies have confirmed that when heart failure occurs, the ventricular volume will increase sharply. With the rapid increase of ventricular end diastolic pressure, the ventricular wall tension will continue to increase, and the natriuretic peptide system will be greatly activated, which will stimulate the secretion of natriuretic peptide by ventricular myocytes, increasing NT-proBNP concetrations.^15^

NT-proBNP levels have become an important indicator for the diagnosis and severity of heart failure, and are positively correlated with the severity of heart failure.^16^ The important aspects of clinical treatment for elderly patients with DVHD and heart failure are diuresis and cardiac strengthening. When the degree of heart failure is effectively controlled, the NT-proBNP level will also be significantly reduced.^17^ At present, clinical studies have shown that the expression of immune factors in serum is also an important cause of heart associated DVHD.^18^ TNF-α can damage vascular endothelial cells, lead to immune adhesion, and cause microthrombosis. At the same time, it can activate a variety of cellular active substances, causing tissue and organ damage.^19^

IL-1β can cause cardiac myocyte hypertrophy, promote fibroblast proliferation, reduce myocardial contractility, and aggravate organ damage.^20^ IL-6 is a multifunctional cytokine, which plays a role in promoting the expression of myocardial cell adhesion molecules, and can effectively enhance the adhesion of myocardial cells and leukocytes, thereby aggravating the damage of myocardial cells.^21^ During the application of metoprolol β-Receptor-1 has a selective blocking effect, which can effectively reduce catecholamine release, improve cardiac output, reduce myocardial oxygen consumption, and promote myocardial remodeling. The application of torasemide can promote the correction of electrolyte disorder in patients with heart failure.^5^ Therefore, the combination of the two drugs can better control the level of NT-proBNP and related inflammatory factors in patients’ plasma.

In this study, the total efficacy of clinical treatment in the Observation-group was as high as 97.01%, significantly higher than 83.87% in the Control-group. In terms of safety, no patients experienced serious adverse drug reactions during treatment. Only a few patients had mild nausea, dizziness, headache and other symptoms. After the drug was stopped, the symptoms improved without special treatment. Therefore, metoprolol combined with torasemide is safe and does not increase adverse drug reactions.

### Limitations:

This was a single center retrospective analysis, small sample size and only 129 cases were included. Therefore, more high-quality, multi center, prospective randomized controlled studies are needed to confirm he results of this study in the future. In addition, there was no patient follow up, and the long-term efficacy and safety of metoprolol combined with torasemide need to be confirmed by further research.

## CONCLUSION

Metoprolol combined with torasemide is safe and can effectively improve cardiac function and reduce NT-proBNP concentrations, resulting in a significant therapeutic effect on DVHD patients with heart failure.

### Authors’ contributions:

**KR** conceived and designed the study.

**JF, XY, BR and YG** collected the data and performed the analysis.

**KR** was involved in the writing of the manuscript and is responsible for the integrity of the study.

All authors have read and approved the final manuscript.
